# Late Replication Domains Are Evolutionary Conserved in the *Drosophila* Genome

**DOI:** 10.1371/journal.pone.0083319

**Published:** 2013-12-31

**Authors:** Natalya G. Andreyenkova, Tatyana D. Kolesnikova, Igor V. Makunin, Galina V. Pokholkova, Lidiya V. Boldyreva, Tatyana Yu. Zykova, Igor F. Zhimulev, Elena S. Belyaeva

**Affiliations:** 1 Institute of Molecular and Cellular Biology, Siberian Branch of the Russian Academy of Sciences, Novosibirsk, Russia; 2 Research Computing Centre, The University of Queensland, Brisbane, St Lucia, QLD, Australia; Duke University, United States of America

## Abstract

*Drosophila* chromosomes are organized into distinct domains differing in their predominant chromatin composition, replication timing and evolutionary conservation. We show on a genome-wide level that genes whose order has remained unaltered across 9 *Drosophila* species display late replication timing and frequently map to the regions of repressive chromatin. This observation is consistent with the existence of extensive domains of repressive chromatin that replicate extremely late and have conserved gene order in the *Drosophila* genome. We suggest that such repressive chromatin domains correspond to a handful of regions that complete replication at the very end of S phase. We further demonstrate that the order of genes in these regions is rarely altered in evolution. Substantial proportion of such regions significantly coincide with large synteny blocks. This indicates that there are evolutionary mechanisms maintaining the integrity of these late-replicating chromatin domains. The synteny blocks corresponding to the extremely late-replicating regions in the *D. melanogaster* genome consistently display two-fold lower gene density across different *Drosophila* species.

## Introduction

Domain organization of the genome has recently become central to our understanding of how eukaryotic genome functions. There are many ways to subdivide a genome into distinct domains and then search for the correlation between the distribution of genes and specific chromatin features. Clearly, domain organization is essential for proper functioning of the genome. However, the very functionality of such domains typically remains untested. One of the parameters that could confirm the functional importance of a specific sequence in the genome is its evolutionary conservation. Recent studies of *Drosophila* genome evolution showed that there are regions where chromosome rearrangement breakpoints tend to cluster, and which have been recurrently used in evolution. On the other hand, there are regions that are virtually never involved in rearrangements [1], [2]. Hence, the question is what is so special about these regions where gene order remains intact across different species?

Earlier it was shown that *Drosophila* genes tend to be clustered into chromatin domains. These domains are characterized by various combinations of chromatin proteins. Domains enriched with histone H1, LAM, SUUR and D1 (markers of repressive chromatin) display higher conservation of gene order between *D. melanogaster* and *D. pseudoobscura* [3]. In a later study comparing nine *Drosophila* species [4], chromatin domains associated with B-type Lamin and SUUR had the lowest probability of being disrupted by rearrangement breakpoints.

Recently, it has been shown that chromatin enriched with histone H1, D1, SUUR and LAM (as well as with four more proteins of unknown functions) represents a widespread type of chromatin, encompassing as much as half of the euchromatic part of *D. melanogaster* genome. It is organized into extensive domains spanning up to hundreds of kilobase pairs [2], [5]. This chromatin (referred to as BLACK chromatin) forms large blocks, harbors silent genes and replicates very late [6]. Little is known about the functional importance of BLACK domains. Thus, the analysis of gene order conservation in such regions could help uncover whether these regions are protected from chromosome rearrangements, in other words, whether their integrity is important or not.

We decided to focus on the conservation of gene order in a number of regions that display the most prominent features of repressive chromatin and replicate the last. About 240 large late-replicating domains were mapped in polytene chromosomes from *Drosophila* salivary glands [7]. About a quarter of these regions fail to complete replication by the end of the S-phase of endocycle. In polytene chromosomes, such regions form constrictions with lower counts of DNA fibers. These regions frequently appear as chromosome breaks [8]–[10] and undergo ectopic pairing between each other, as well as with pericentric heterochromatin. This is likely a consequence of repair-mediated end-joining of double-stranded DNA breaks in underreplicated regions [11]–[13]. These “weak spots” and ectopic pairing were originally established as the cytological markers of underreplicated regions.

As early as in 1939, underreplicated regions appearing in polytene chromosomes as large dense bands were called intercalary heterochromatin (IH) [14]: morphologically they looked similar to the classic pericentric heterochromatin. However, in contrast to pericentric heterochromatin, IH domains are not enriched in repeated DNA sequences. They are typically composed of unique genes scattered throughout the regions at a lower than the genome average density [15], [16]. One of the molecular markers of late replication is SUUR protein. SUUR levels modulate underreplication [17]–[19] by decreasing replication fork progression rate [20].

Genome-wide mapping of IH regions was performed using underreplication as a marker [15], [19]. Underreplicated regions thus far identified span several hundreds kb and comprise up to several dozens of genes. Despite the fact that borders of IH regions could only be approximately inferred from the local levels of underreplication, these studies allowed comparison of chromatin organization in polytene chromosomes and in chromosomes from mitotically dividing cells. The level of underreplication in polytene chromosomes was found to be positively correlated with SUUR enrichment in chromosomes of embryonic Kc cell line [21], [22]. Thus, IH regions represent a special class of chromatin domains that are formed not just in polytene chromosomes, but in chromosomes of proliferating cells, as well.

Accurate mapping of the borders of these chromatin domains only became possible when modENCODE project data have become available [http://www.modencode.org]. It was shown that several proteins specific for the interbands of polytene chromosomes invariably mapped to the same regions in chromosomes of diploid cells [23]. Thus, the distribution of these proteins [6], [24], [25] marks the borders of IH regions in polytene chromosomes. Sixty such IH regions have been accurately mapped to date [22]. These 60 regions are hereafter referred to as UR(B)-regions. They turned out to correspond to large domains of BLACK repressive chromatin [6].

It must be emphasized that in addition to these 60 IH regions, a handful of sites also demonstrate “weak spots” and underreplication, albeit to a lesser extent [7], [19]. These sites still await accurate mapping, so in this paper we only analyze UR(B)-regions.

Besides underreplicated regions, in salivary gland polytene chromosomes there are many other domains of late replication; they are marked with SUUR, yet display no underreplication. Grouping the regions into underreplicated and late-replicating subsets is somewhat arbitrary, because underreplication is restricted to polytene chromosomes, it is dependent on *SuUR* gene dosage [7] and is tissue-specific [19]. We will nevertheless keep this classification to facilitate data presentation.

Knowledge of exact borders of UR(B)-regions allowed the detailed analysis of their features at the molecular level. These regions were established as extensive domains of repressive chromatin that are virtually devoid of replication origins both in salivary gland chromosomes and in cell lines [6], [20], [22], [26]. Absence of internal origins of replication, large size and stalling of replication fork in the presence of SUUR – all these factors contribute to DNA underreplication in polytene chromosomes. UR(B)-regions are known to house many tissue-specific genes, which require complex regulation of expression. So one can expect these peculiar domains to be conserved in evolution, i.e. that these regions are “cold spots” for chromosome rearrangement breakpoints.

In this work, we show that in contrast to other genes, late-replicating genes in BLACK chromatin tend to preferentially keep the linkage with their neighbors. Then we proceed to explore the conservation of gene order in UR(B)-regions and conclude that they are rarely broken by chromosomal rearrangements and frequently correspond to large synteny blocks. Conversely, large synteny blocks also tend to map to UR(B)-regions. We can thus link the regions with conserved gene order to specific chromatin domains with known borders. This allowed a more comprehensive analysis of these regions at the domain level, rather than on a gene-by-gene basis, as was performed previously [4]. As a result, we show that high IGA-scoring regions typically harbour repressed chromatin; they display narrow temporal expression pattern, extra-late replication and low gene density. Notably, we show that the latter feature tends to be present across different *Drosophila* species, which suggests the evolutionary conservation of both gene order and chromatin status.

## Results

We analyzed the evolution of gene order in repressive late-replicating domains of *Drosophila* genome. As a measure of conservation, we used «orthologous landmarks» (OLs) from the work of Grotthuss and colleagues [2]. OLs were identified upon comparison of 9 *Drosophila* species: *D. melanogaster*, *D. erecta*, *D. yakuba*, *D. ananassae*, *D. pseudoobscura*, *D. willistoni*, *D. virilis*, *D. mojavensis* and *D. grimshawi*. To build OLs, the authors used independent gene anchors (IGAs): each of the IGAs corresponded to a single gene or to a group of physically linked (overlapping) genes, and was considered as a single evolutionary unit [2]. Every OL is represented either by a set of IGAs, whose order (for orthologous genes) remains intact across nine *Drosophila* species or by an individual IGA found in all the species analyzed. Three ways to subdivide the genome into OLs were proposed, depending on the stringency of synteny definition [2].

Our results were obtained on synteny blocks defined by a GO criterion requiring conservation of Gene Order regardless of gene orientation, and were reproduced using other definitions of synteny blocks based on the conservation of Gene Order and Orientation (GOO), as well as using Overall Local Contiguity (OLC) dataset where gene scrambling within an OL is permitted.

### Genes from multigenic OLs display late replication timing and mainly map to repressive regions of BLACK chromatin

**Figure 1:**
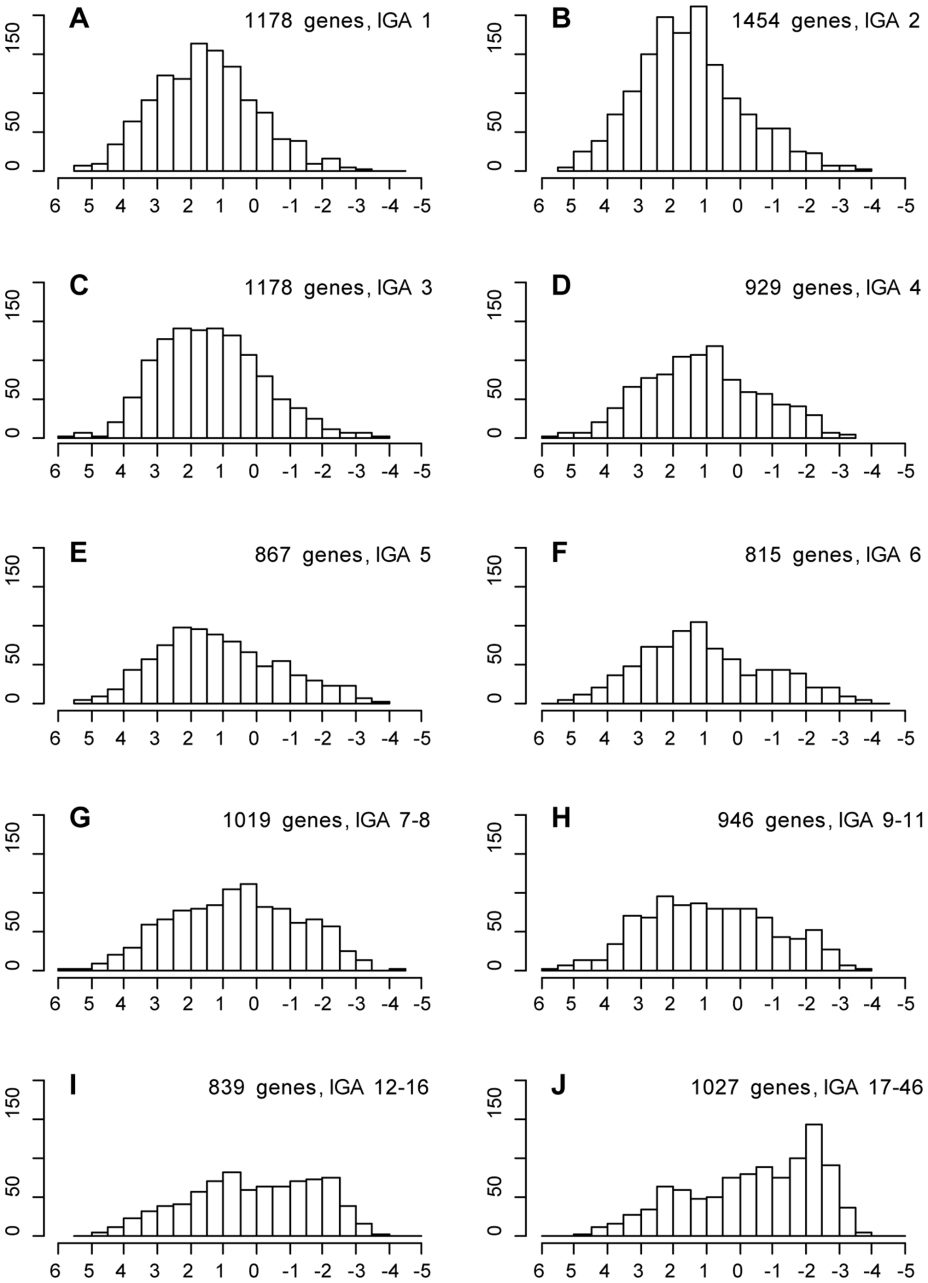
Replication time in Kc cells for genes in OLs with different IGA scores. Replication score is shown on the *X* axes, with +6 corresponding to early replication and −5 to the late replication. Gene counts are shown on the *Y* axes.

First, we wanted to test the hypothesis that genes residing in multigenic OLs tend to replicate late in the S phase. To do so, we used genome-wide replication profile from *D. melanogaster* Kc cell line [27]. For each gene, replication time was averaged across all the corresponding probes (see further details in Materials and Methods). IGA score was calculated for each gene as the number of IGAs within the appropriate OL. Next, genes with identical IGA scores were combined together. Similar-sized groups were formed from genes with high IGA scores. Fig. 1 illustrates that genes from high IGA-scoring OLs display overall later replication. To estimate the statistical significance of the observed difference in the replication timing, we randomly selected the same number of genes for every group (Fig. 1) and calculated the mean replication timing score of the shuffled genes. To account for the similarity in replication timing between adjacent genes, we maintained the syntenic structure in the shuffling (see details in Materials and Methods). For each group the shuffling was repeated 10,000 times. Difference between the observed and expected mean replication timing is statistically significant (P-value<E-4) for genes with low and high IGA score (Table S1). Similar trends were observed when using replication profiles obtained for *D. melanogaster* Cl8 cell line [27] (Fig. S1; Table S2), which lends further support to the idea that later replication is characteristic of the regions with conserved gene order.

It was shown that in Kc cells chromatin domains defined by specific sets of proteins displayed distinct replication timing [6]. Repressive BLACK chromatin is the last to undergo replication, whereas active YELLOW chromatin, encompassing predominantly house-keeping genes, replicates early. Accordingly, taking into account that late replication appears linked with high IGA-scoring OLs, BLACK chromatin domains would be expected to display higher conservation of gene order, whereas YELLOW chromatin would be less conserved.

**Figure 2:**
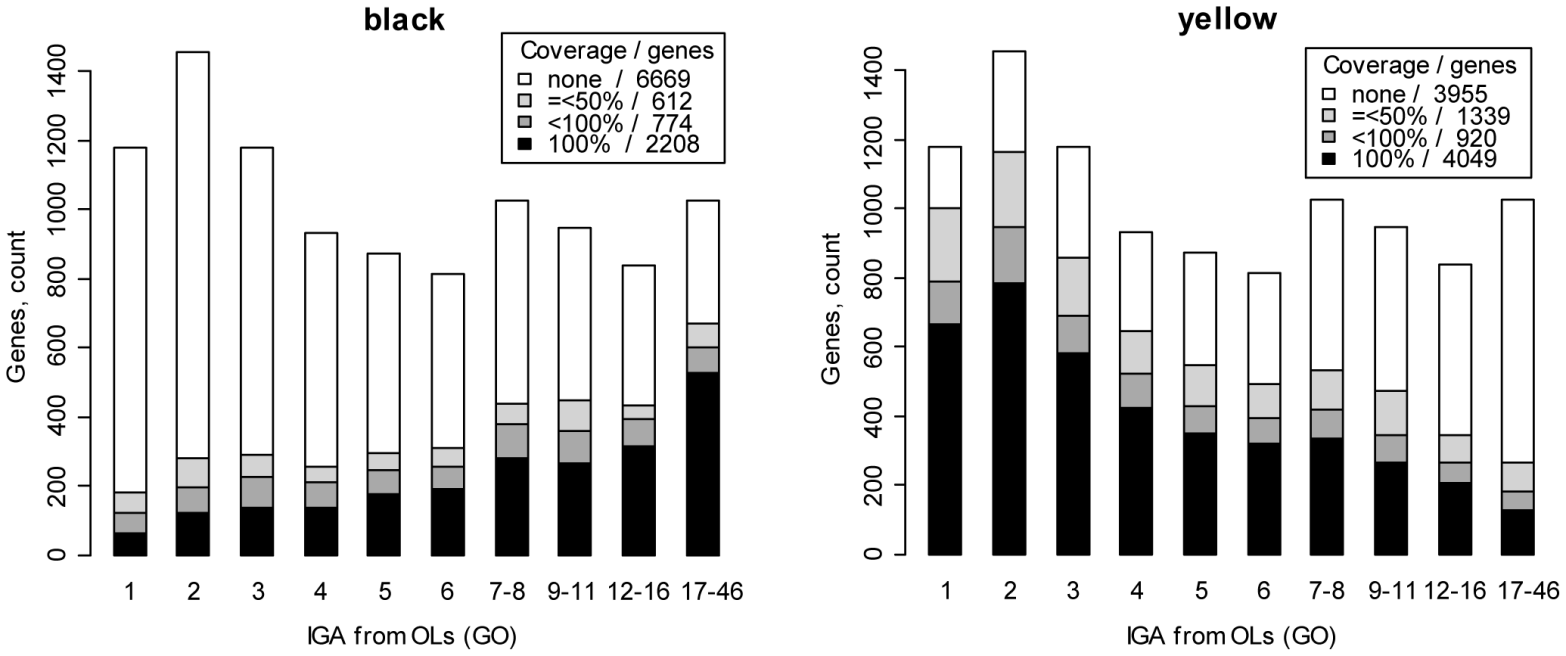
IGA score for genes covered by BLACK and YELLOW chromatin. *X* axis shows the number of IGAs within OLs assigned to genes; gene counts are shown on the *Y* axis.

To test this idea, we analyzed whether IGA score correlated with repressive BLACK or active YELLOW chromatin types. Figure 2 shows that there are two pronounced trends: BLACK chromatin is quantitatively enriched with multigenic OLs, whereas YELLOW chromatin is generally composed of oligogenic OLs (for statistics, see Table S3). Out of 1,178 genes with IGA score 1, only 121 (10%) have >50% of their base pairs covered by BLACK chromatin compared to 600 out of 1027 (58%) genes with IGA score 17–48 (2×2 contingency table, the Yates Chi [2] 575.86, P-value 3E-127). By contrast, YELLOW chromatin covers more than 50% of base pairs in 787 genes (67%) with IGA score 1, but it is found only in 182 genes (18%) with IGA score 17–48 (2×2 contingency table, the Yates Chi [2] 534.68, P-value 3E-118). We therefore suggest that chromatin domains showing late replication are the zones with conserved gene order.

### UR(B)-regions are characterized by low level of synteny breaks

Having established that late-replicating genes are predominantly found in the regions with higher degree of gene order conservation, we focused our analysis on a set of 60 regions replicating very late in salivary glands [22] (see Introduction for more details). These 60 UR(B)-regions cover 14.8 Mb (12.3%) of the euchromatic part of the *D. melanogaster* genome but overlap with just 106 OLs (4%). In order to estimate the statistical significance, we shuffled these 60 regions in the genome using BEDTools shuffleBed [28] and counted the overlapping OLs. In 100,000 shuffling iterations, the smallest number of OLs overlapping the “randomized” set of UR(B)-regions was 235, indicating that the observed number of overlaps is significantly lower than expected by chance (P-value<1E-5). On average, the “randomized” UR(B)-regions overlapped 357 OLs, 3.4 times more than the observed value. A similar trend was found using other definitions of synteny [2] (see Text S1).

**Figure 3:**
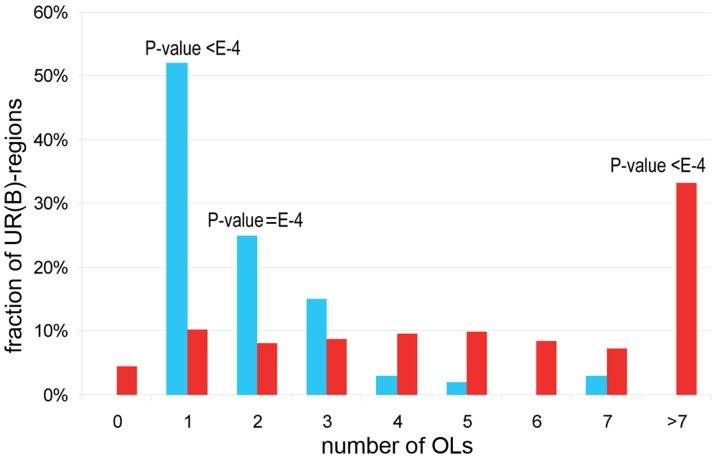
Overlap between OLs and UR(B)-regions. *X* axis shows the number of OLs that overlap with a single UR(B)-region, *Y* axis shows the percentage of the corresponding UR(B)-regions in the total set of UR(B)-regions. Blue bars indicate the numbers observed for actual UR(B)-regions. Red bars correspond to the simulated counts obtained for a randomly shuffled set of UR(B)-regions via 100,000 shuffling iterations. P-values are provided on top of the bars when differences between the observed and expected values reach statistical significance.

While the number of OLs overlapping any UR(B)-region ranges from one to seven, 31 (52%) UR(B)-regions overlap with just a single OL (Fig. 3), 5.1-fold more than the average 6.1 observed in the shuffled control (100,000 iterations, P-value<1E-5). The number of UR(B)-regions overlapping with just two OLs is also higher than expected. By contrast, significantly fewer UR(B)-regions overlap with multiple OLs (Fig. 3).

Out of 60 UR(B)-regions, 41 (68%) have at least 80% of their length covered by a single long OL, 4.7-fold more than expected (shuffling, 100,000 iterations, average 8.8, maximal number of regions with at least 80% coverage - 22, P-value<1E-5). This result shows that most UR(B)-regions have significant proportion of their length covered by a single synteny block.

**Figure 4:**
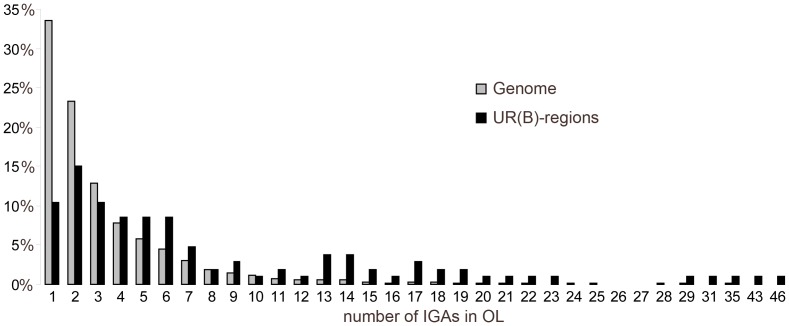
Distribution of OLs with different IGA scores across the genome and in UR(B)-regions. IGA counts per OL are shown on the *X* axis. *Y* axis shows the percentage of the corresponding OLs in the genome (grey) and in the UR(B)-regions (black).

OLs overlapping UR(B)-regions have more IGAs. Out of 106 OLs that overlap with UR(B)-regions, there are 50 OLs (47.2%) with IGA scores 5 or higher. This number of high IGA-scoring OLs is 2.8 times more than the expected number of 17.7 (Chi [2] test, P-value = 3.5E-17). In UR(B)-regions, the proportion of OLs with an IGA score 1 is less than 10%, compared to 35% observed for the entire genome. By contrast, the fraction of multigenic OLs is significantly higher in UR(B)-regions than across the genome (Fig. 4, Fig. S2). The average IGA score of OLs in the genome is ∼3.4 (median 2), whereas OLs located in UR(B)-regions have an average IGA score of 8.3 (median 5). Thus, OLs in UR(B)-regions have higher IGA scores compared to the genome-average (Chi [2] test, P-value<1E-6).

Our results demonstrate that majority of UR(B)-regions overlap multigenic OLs. More than half of UR(B)-regions overlap with just one OL. Furthermore, 68% of UR(B)-regions have at least 80% of their sequence covered by a single OL indicating that UR(B)-regions have fewer synteny breaks compared to the expectation based on the random distribution of UR(B)-regions in the genome. At the same time, the proportion of multigenic OLs is higher in UR(B)-regions compared to the genome average.

Unfortunately, the exact positions of synteny breakpoints between OLs are not known. We used midpoints between neighboring OLs as a proxy for synteny breaks. Out of 60 UR(B)-regions, 24 (40%) do not overlap with such “synteny breaks”, which is 3.2 times less than observed in the shuffled control (100,000 iterations, maximal number of regions without the breaks - 21, P-value<1E-5). In addition, 20 UR(B)-regions (33%) overlap with just one “synteny break”, 3.9-fold less than observed in shuffling (100,000 iterations, maximal number of regions with one break - 17, P-value<1E-5).

### Many UR(B)-regions coincide with OLs

**Figure 5:**
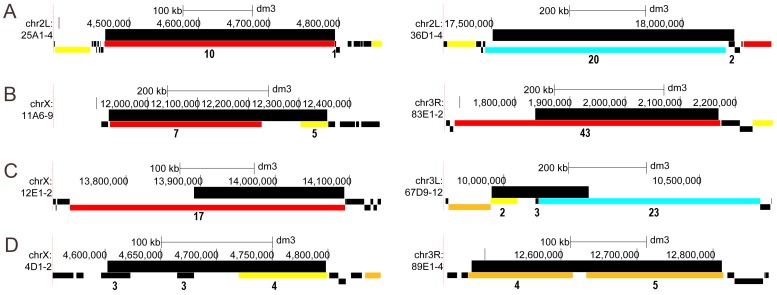
Examples of different types of overlap between UR(B)-regions and OLs. Different scales are used for each region. Wide black box denotes an UR(B)-region (name is shown on the left). Narrow colored boxes below correspond to OLs (black: <50 kb; yellow: 50–100 kb; orange: 100–200 kb, red: 200–500 kb, blue: >500 kb). The IGA score is shown under each OL overlapping the UR(B)-region. For each type of overlap two examples are shown. **A** - nearly exact correspondence between UR(B)-region and long OL, with reciprocal overlap over 80%; **B** – reciprocal overlap ranges 65–80%; **C** – UR(B)-region overlaps with a large OL, but the extent of overlap is below 65%; **D** – UR(B)-region overlaps with several smaller OLs, neither of which appears to be dominant length-wise.

A significant proportion of UR(B)-regions almost entirely coincide with OLs. Fig. 5 illustrates relative positions of several groups of UR(B)-regions and OLs (all regions are shown in Fig. S3). To quantify the extent of this match, we selected UR(B)-OL pairs with greater than 80% reciprocal overlap. Out of 60 UR(B)-regions, 17 have nearly perfect overlap with a single long OL: at least 80% of UR(B)-region sequences are covered by a single OL, and, vice versa, the UR(B)-region covers at least 80% of the corresponding OL. In 100,000 shuffling iterations the average number of UR(B)-OL pairs with at least 80% reciprocal overlap was 2.1, 8.1 times less than the observed value, and the highest number was 12, consistent with a P-value below 1E-5. Notably, the UR(B)-region 50C shows 79.8%/93.3% reciprocal overlap with an OL (just slightly below the 80% cutoff), which further increases the proportion of UR(B)-regions significantly overlapping with OLs. Similar trend was observed using other definitions of synteny [2] (see Supplementary text S1 for details).

The proportion of UR(B)-regions showing nearly perfect overlap with OLs is even higher if we consider closely located UR(B)-regions. In 7 cases, pairs of UR(B)-regions are separated by fairly small regions of active chromatin ranging from several kb to several dozens kb, but both UR(B)-regions in a pair are covered by the same OL. Out of 7 such pairs, 6 have reciprocal overlap with a single OL for at least 80% of their length. If we consider such pairs as a single region, 45% of UR(B)-regions would have at least 80% reciprocal overlap with a single OL. This supports our idea that significant proportion of UR(B)-regions are cold-spots for chromosomal rearrangements, as compared to the total genome where synteny breaks on average happen more frequently.

### Ultraconservative OLs map to the late-replicating regions of polytene chromosomes

We showed that OLs overlapping UR(B)-regions had on average more genes (IGAs) compared to the OLs across the entire genome. We then tested whether the opposite was also true, i.e. whether OLs with the highest IGA counts in the *Drosophila* genome were enriched among UR(B)-regions. Von Grotthuss et al. [2] identified 22 OLs encompassing over 20 IGAs, and termed them ultra-conserved regions (UCRs). Just 8 out of 22 UCRs co-localize with UR(B)-regions, not much different from an average value obtained in the shuffled control. In order to test if any of the remaining UCRs map to the late-replicating regions, we mapped the probes from these regions on polytene chromosomes using FISH. For every region, we designed a FISH probe within its central part and hybridized it to salivary gland polytene chromosomes (Fig. S4). Table 1 summarizes cytological mapping data obtained for all such probes. 13 out of 22 UCRs (∼59%) were observed to map to the underreplicated regions, which were documented to have weak spots, late replication sites and SUUR binding. 6 UCRs (27%) match the positions of late-replicating regions bound by SUUR. Three UCRs (14%) do not coincide with the regions showing late replication. Thus, the vast majority of UCRs are found in the regions of late replication, and over half of UCRs map to underreplicated regions.

**Table 1:** Localization and properties of UCRs.

	#OL	Number of IGAs	Genomic location (genome 5 release)	Size (bp)	Cytology location (polytene band)	Relation to UR-regions
1	98	24	chrX:2628361-3026836	398476	3C3-5*	UR
2	598	22	chr2L:1170235-1502903	332669	22A1-3*	UR
3	652	25	chr2L:3530904-3784129	253226	24A1-2*	-
4	713	29	chr2L:6127867-6455934	328068	26C1-2	UR(B)
5	808	28	chr2L:9934750-10202233	267484	31A1-2*	UR
6	872	21	chr2L:13914726-14328265	413540	34F1-4*	UR
7	1025	29	chr2R:2892724-3110885	218162	43A1-2*	LR
8	1354	24	chr2R:14072292-14327986	255695	55C1-5*	LR
9	1384	28	chr2R:15395673-16100124	704452	56F**	LR
10	1430	22	chr2R:17580236-17863710	283475	58A3-4	UR(B)
11	1471	21	chr2R:18968085-19242753	274669	59D1-4	UR(B)
12	1669	46	chr3L:4407146-4996877	589732	64C1-2; 64C3-4	UR(B)
13	1693	25	chr3L:6068660-6166966	98307	65A1-6*	LR
14	1717	29	chr3L:7362900-7633601	270702	66A1-2*	LR
15	1744	27	chr3L:8617474-8925165	307692	66E1-2*	-
16	1776	23	chr3L:10088848-10656077	567230	67D9-12	UR(B)
17	1893	35	chr3L:16225132-16340720	115589	72E1-2*	LR
18	2106	43	chr3R:1690872-2173240	482369	83E1-2	UR(B)
19	2124	35	chr3R:3073872-3622321	548450	84D3-4;84D9-10	UR(B)
20	2533	26	chr3R:20159146-20356280	197135	96A1-2*	UR
21	2598	22	chr3R:22811389-22937977	126589	97E1-2*	-
22	2665	31	chr3R:26407921-26762229	354309	100A1-2; 100B1-2	UR(B)

FISH mapping performed in the present paper.

FISH-based FlyBase mapping data in wild type chromosomes (for further details see Materials and Methods).

LR – late-replicating region.

UR – underreplicated region.

### OLs in UR(B)-regions are among the longest in the genome

OLs overlapping UR(B)-regions tend to be long. The average and median lengths of OLs overlapping UR(B)-regions are 150 kb and 93.1 kb, respectively, 4.1- and 7.5-fold higher than the genomic average 36.3 kb or median 12.5 kb.

**Figure 6:**
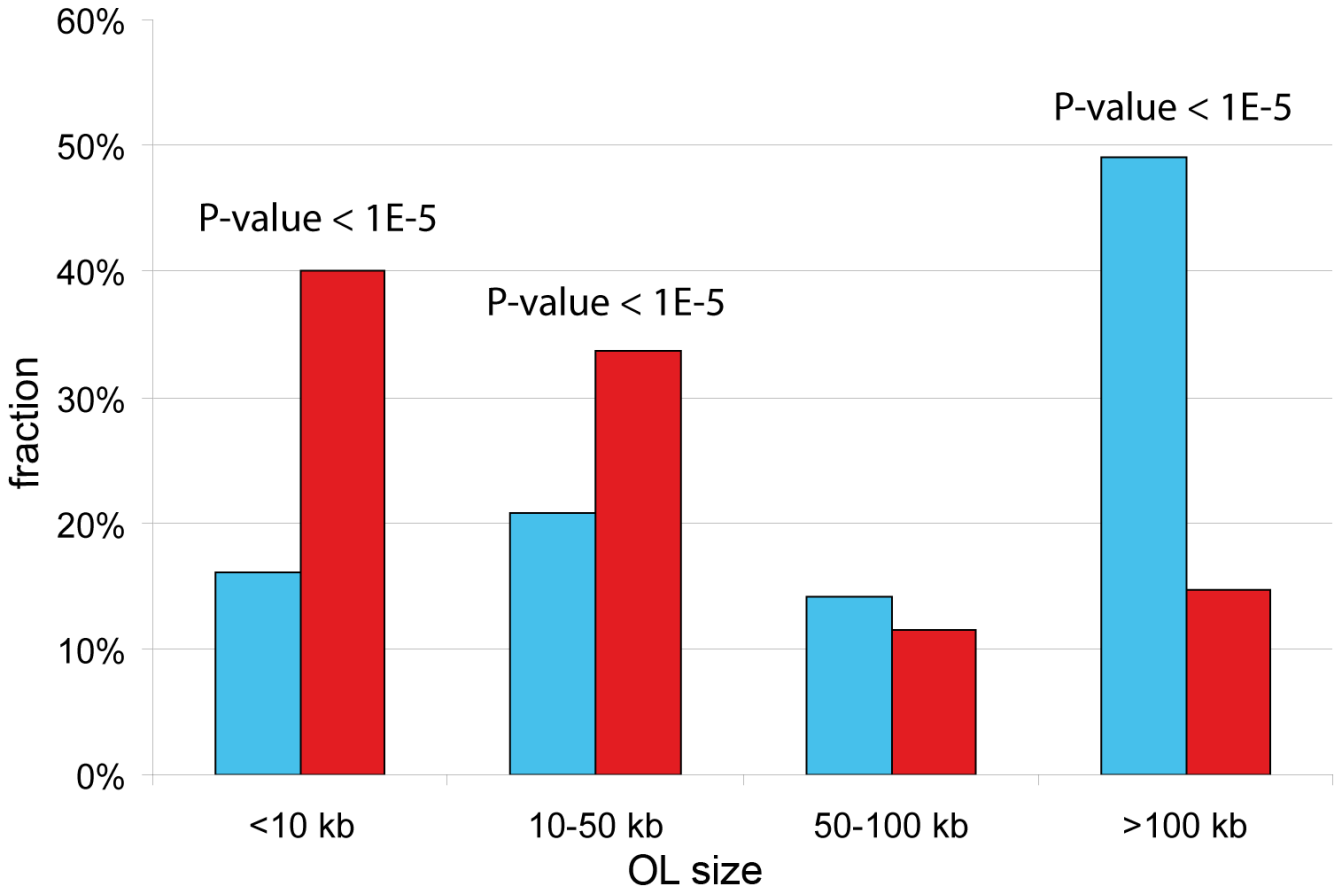
Comparison between the observed and simulated distributions of OL lengths in UR(B)-regions and across the genome. OL length is shown on the *X* axis. The observed percentage of OLs of a given size in UR(B)-regions is shown as blue. Red bars denote an average value obtained for a randomly shuffled set of UR(B)-regions by 100,000 shuffling iterations. P-values are shown on top of the bars where statistically significant differences between the real and expected values are achieved.

The fraction of OLs below 10 kb is 2.5-fold lower in UR(B)-regions as compared to the randomly shuffled UR(B)-regions (16.0% vs 40.1%, range 28.4%–54.6%, P-value<1E-5) (Fig. 6). Likewise, OLs ranging 10–50 kb are underrepresented in UR(B)-regions (20.8 vs 33.7, range 23.3%–43.1%, P-value<1E-5). OLs ranging 50–100 kb show no statistical difference with shuffled values (14.1% vs 11.5%). However, there are 3.3-fold more OLs spanning over 100 kb in UR(B)-regions, as compared to the expected value (49.1% vs 14.7%) (Fig. 6). Thus, OLs within UR(B)-regions have large physical size. The same trend for longer OLs within UR(B)-regions was observed using OLC and GOO definitions (Text S1).

Out of 30 longest OLs found in the *Drosophila* genome, 18 OLs (60%) overlap with UR(B)-regions. In 100,000 iterations, on average only 8.5 longest OLs overlapped with randomly shuffled UR(B)-regions, 2.1-fold less that the observed value (P-value = 9E-5), and the remaining 12 longest OLs were co-localized with late-replicating regions and/or regions stained by SUUR antibodies (Table 2). We conclude that the longest OLs in the genome are biased toward UR(B)-regions.

**Table 2:** Cytology positions of the largest OLs.

	#OL	Size (bp)	IGA value	Cytology location	Relation to the UR-regions
1	1384	704452	28	56F**	LR
2	921	630503	20	36D1-4	UR(B)
3	1669	587980	46	64C1-2; 64C3-4	UR(B)
4	1776	567230	23	67D9-12	UR(B)
5	2124	548450	35	84D3-4; 84D9-10	UR(B)
6	1948	514362	19	75C1-2	UR(B)
7	551	487465	18	19E1-2; 19E3-4	UR(B)
8	2106	482369	43	83E1-2	UR(B)
9	852	418522	17	34A1-2	UR(B)
10	872	413540	21	34F1-4*	UR
11	98	398476	24	3C1-5*	UR
12	2620	383126	13	98C†	UR
13	2414	379580	14	92A**	LR
14	395	369210	17	12E1-2	UR(B)
15	1871	360550	12	71C1-2	UR(B)
16	2665	354309	31	100A1-2; 100B1-2	UR(B)
17	996	352347	13	40C‡	UR
18	830	347613	13	33A** †	UR
19	1839	346113	14	70A1-2; 70A4-5	UR(B)
20	212	338653	9	7B1-2	UR(B)
21	791	335570	16	30A**	UR
22	598	332669	22	22A1-3*	UR
23	1857	330850	11	70D**	LR
24	655	330344	12	24D** †	UR
25	671	328842	10	25A1-4	UR(B)
26	713	328068	29	26C1-2	UR(B)
27	2276	324929	18	87D1-2	UR(B)
28	2222	324302	17	86D1-2	UR(B)
29	1315	315415	14	54AB**	LR
30	1847	313407	2	70C1-2	UR(B)

FISH mapping performed in the present paper.

FlyBase FISH mapping data in wild type chromosomes (further details in Materials and Methods).

Mapping position was deduced based on the position of the corresponding underreplication zone, according to [19].

Mapping position was established according to the matching underreplication zone referenced in [15].

LR – late-replicating region.

UR – underreplicated region.

While on average UR(B)-regions have OLs with more IGAs than is found throughout the genome (see above), not all UR(B)-regions coincide with high-IGA OLs. For instance, the region 70C1-2 shows almost perfect overlap with OL #1847, which has just 2 IGAs, yet spans 313 kb. Similar examples include the regions 33A1-2, 77E1-4 and 92D1-4, which map to OL# 831 (6 IGAs, 221 kb), # 1995 (7 IGAs, 241 kb) and #2424 (7 IGAs, 188 kb), respectively. Such a combination of big length and low IGA counts within synteny blocks reflects low gene density that is characteristic of underreplicated regions [16].

### UR(B)-regions found in D. melanogaster display similar features in other Drosophila species

Lower gene density was shown to be a good predictor of underreplicated region localization in *D. melanogaster* [16], so we explored whether this was also observed for other *Drosophila* species. Indeed, substantial overlap between UR(B)-regions and OLs is indicative of the preservation of such regions in other species. From the data on OL localization to UR(B)-regions in *D. melanogaster*, we can readily estimate gene density in these same OLs in other species. Lower gene density within OLs would indirectly support the idea that the properties of underreplicated regions are maintained within OLs throughout evolution.

To estimate gene density in non-melanogaster *Drosophila* species, we adapted the approach described by [16], where gene density was compared within and immediately outside of underreplicated regions. For this analysis, we selected the OLs that displayed greater than 80% overlap with UR(B)-regions (pairs of UR(B)-regions covered by a single OL were considered as a single region). We removed OL regions whose flanking sequences overlapped with the neighboring UR(B)-regions. In total, there were 18 OLs overlapping with 23 UR(B)-regions, including 5 “split” OL regions (see Materials and Methods). Gene density in these selected *D. melanogaster* OLs was 2.2 times lower than in the flanking regions (Table S4, one-tailed paired ttest, P-value = 1.6E-7), which is in good agreement with the previously published data [16].

**Figure 7:**
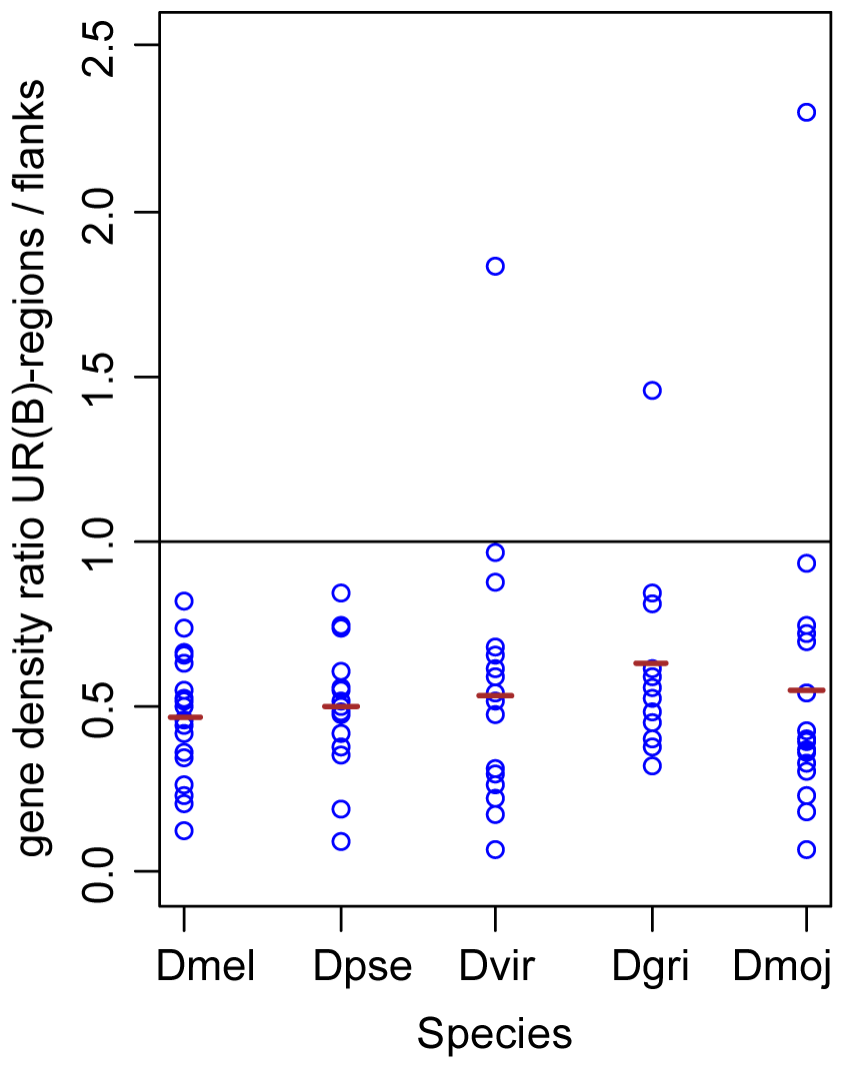
Ratio of gene density in OLs overlapping UR(B)-regions and in their immediate flanking regions observed in several *Drosophila* species. *Dmel = D. melanogaster*, *Dpse = D. pseudoobscura, Dvir = D. virilis*, *Dmoj = D. mojavensis*, *Dgri* = *D. grimshawi*. OLs with a mutual overlap with UR(B)-regions of 80% or greater were used for this analysis. Each circle represents the ratio gene density in an individual OL and in its flanks. Red horizontal lines denote average ratio values calculated for the entire sampling of OLs in each species. These values are all clustered around 0.5, i.e. gene density in OLs in on average twice as low as in OL flanks.

In *D. pseudoobscura* and *D. virilis*, gene density in 18 OLs corresponding to the *D. melanogaster* UR(B)-regions is 2.1- and 2.2-fold lower compared to the flanks (one-tailed paired ttest, P-value = 1.4E-6 and 2.3E-5). Only 13 OLs corresponding to the *D. melanogaster* UR(B)-regions have satisfactory assembly quality in the *D. grimshawi* genome (gene density is 1.7-fold lower than in the flanks, one-tailed paired ttest, P-value = 3.7E-4). The same is observed for 17 OLs in the *D. mojavensis* genome: gene density is 2.3-fold lower compared to the flanks, one-tailed paired ttest, P-value = 9.6E-5. In all of these species, gene density in OLs corresponding to *D. melanogaster* UR(B)-regions is roughly twice as low as in the flanking sequences (Fig. 7, Table S4). Thus, we can conclude that low gene density is a property of these regions that is conserved throughout evolution.

## Discussion

Until recently, synteny could only be studied at the level of large chromosomal blocks. There are numerous studies exploring synteny in mammals, however, as a rule synteny blocks of small sizes were omitted from these analyses, and conclusions were typically based on the data with resolution on the order of megabase pairs [29, and references therein]. Resolution threshold varied from paper to paper, and this circumstance dictated the outcome of analysis [30]. Despite this, the common theme for mammalian genome studies was the presence of cold and hot spots of chromosomal rearrangements [31]–[33]. The zones that were frequently hit by chromosomal rearrangements throughout evolution were typically gene-rich [34]–[36], had higher frequency of segmental duplications and/or repetitive elements [33], [37]–[39], and were frequently associated with chromosome fragile sites [40]. In terms of their genetic functions, these regions were enriched for genes associated with adaptation. At the same time, rearrangement cold spots, where the order of genes remained fixed for millions of years, were found to be significantly enriched for genes involved in development of the central nervous and other organ systems [29].

Availability of 12 sequenced *Drosophila* species with high-quality genome annotation within one genus [41] allows analysis of synteny at significantly greater resolution, i.e. at a gene-level accuracy. In addition, small *Drosophila* genome is very well characterized in terms of chromatin composition. Extensive annotation of *Drosophila* genome made it possible to draw parallels between chromatin, gene organization and gene order conservation.

It was thus demonstrated that genes residing in large OLs were predominantly targeted by SUUR and LAM, – these proteins were established to locate at the nuclear periphery. De Wit et al [3] also reported the domains defined by H1, LAM, SUUR and D1 binding as having fewer synteny breaks than expected. Taking these data together, we suggest that large OLs may correspond to the domains of late replication, also known to be bound by these proteins. Such domains have been extensively studied by our group, and 60 of them replicating the latest were mapped both by cytology and molecular means (see Introduction). This allowed for comparison of domain borders with the positions of OLs. Extensive overlap between the domains of late replication and large OLs was thus demonstrated:

*i)* 41 out of 60 UR(B)-regions (68%) display at least 80% sequence overlap with one large OL; *ii)* over half of UR(B)-regions are matched by a single OL; *iii)* vast majority of UCRs (19 of 22) and top 30 longest OLs overlap with UR(B)- or late-replicating regions.

The total span of 60 UR(B)-regions studied here is 14.8 Mb, which overlaps with 22% of BLACK chromatin sequence [6], [22]. Thus, our results describe a substantial proportion of repressive late-replicating chromatin in the genome. Yet, one may ask whether this feature of evolutionary conservation is also characteristic of other BLACK chromatin regions showing late replication. Our extended genome-wide analysis in *D. melanogaster* shows that there is indeed a significant positive correlation between localization of genes to large OLs, their repressive state and late replication.

Apparently, the links between OLs, repressive chromatin and late replication established here also hold true for the genomes of other *Drosophila* species. This is supported by the fact that low gene density – a peculiar feature of organization of underreplicated regions [16] – is also observed for OLs from 4 more *Drosophila* species. These observations indirectly support the idea that in course of evolution OLs not only keep their gene order, but also maintain the characteristic repressive chromatin status.

Conservation of gene order and repressive state of chromatin may be causally linked. For instance, the frequency of chromosomal rearrangements may be reduced due to stronger compaction of repressive chromatin and/or due to as yet poorly explored mechanisms underlying formation and maintenance of the closed state of BLACK chromatin. One such mechanism may involve binding of repressive proteins at a pre-defined set of “entry sites” with further spreading throughout the entire domain, – as exemplified by HP1-dependent silencing [42].

Maintenance of gene order conservation in underreplicated and late-replicating regions may also be explained by the presence of intergenic regions encompassing particular regulatory sequences, such as highly conserved non-coding elements (HCNEs) required for proper gene expression [43 and references therein]. Multigenic OLs are HCNE-rich, as it was shown for 5 *Drosophila* species [43]. Accordingly, repressive BLACK chromatin was demonstrated to be enriched with HCNEs [6]. Sahagun and Ranz [44] further extended and reinforced this conclusion by analyzing 9 *Drosophila* species. Not only did the authors show the enrichment of OLs with HCNEs, but they also provided the basic “functional portrait” of conserved regions behaving as regulatory domains. HCNE peaks were found in 123 OLs. Putative HCNE targets are tightly associated with specific promoter motifs; compared to other genes, they display higher incidence of severe mutant phenotypes and stronger expression profiles during important developmental transitions. It must be underlined that according to Ranz et al. [4] phylogenetic conservation of gene order is unrelated to lower recombination rate or local co-expression of genes residing within OLs. One explanation for this is that when HCNE-dependent regulation of essential genes is disrupted by chromosomal rearrangements, such events should be negatively selected. However, when an UCR #1384 encompassing 4 HCNE peaks was split by a rearrangement, no significant transcriptional changes were observed [45]. This specific case should not establish a rule, because only a single rearrangement breaking a single region was analyzed.

DNA repair may serve as one of the factors contributing to the peculiar evolution of late-replicating regions. The dynamics of various repair mechanisms throughout the cell cycle has been proposed to explain the increased mutation rate of late-replicating genes in human [46] and *Drosophila* [47] cells, as well as the pronounced association of *Drosophila* late-replicating genes with duplication hotspots [48], [49]. In drosophila, vast (up to 700 kb) regions of late replication lack internal replication origins [20], [22], so converging replication forks have to move long distances until they meet. This peculiar property of replication may increase the mutation rate and thereby result in the accumulation of mutations in these regions. These data argue in favor of higher rates of neutral evolution in the regions of late replication. Yet, very little is presently known about the dynamics of repair components throughout the cell cycle [49], so it is possible that the peculiarities of repair in late-replicating regions may influence the maintenance of gene order conservation.

It has recently been reported that gene pairs with short intergenic distances tend to have higher rearrangement rates as compared to the wider spaced genes [50]. Gene-dense regions of YELLOW chromatin encompassing house-keeping genes are typically localized on the borders of late-replicating regions [22]. It is tempting to speculate that these fragments of YELLOW chromatin serve as hot spots for chromosomal rearrangements, thereby shielding the regions of late replication.

To conclude, we demonstrate that extensive regions of repressive chromatin in the D. melanogaster genome display very late replication and show conserved gene order. Notably, these regions tend to exist in repressed chromatin status in other drosophila species. The reasons underlying this intriguing conservation of gene order are poorly explored and prompt further research.

## Materials and Methods

### Correlation of replication timing and numbers of IGAs per OL

Gene loci were defined as genomic intervals between the leftmost Start and rightmost End positions of all transcript variants for every FlyBase 5.12 gene model. FlyBase Genes 5.12 annotation was downloaded from the UCSC Genome Browser web site. Genes with the CG IDs identical to the genes in the syntenic blocks [2] were used in subsequent analysis.

The data for replication timing in diploid Kc and Cl8 cells [27] were downloaded from ReplicationDomain site: http://www.replicationdomain.com/. The data for chromosomes X, 2 and 3 were filtered by *awk* and four columns (chr, start, end, replication score) were extracted. The resulting BED file was uploaded to Galaxy web site and intersected with FlyBase Genes 5.12 loci using *Join* command from *Operate on Genomic Intervals* menu with requirement of at least 5 bp overlap. The average replication time (RT) for all probes overlapping every locus was calculated by *Group* command in *Join, Subtract and Group* menu. Statistical significance of difference in RT between genes with different IGA scores (Figure 1) and randomized genes was calculated in R package. In order to compensate for the correlation in RT for adjacent genes, we shuffled genes maintaining the syntenic organization, *e.g.*, if the group contains a syntenic block with ten genes, we selected ten consecutive genes (ordered by the genomic position) starting from a randomly chosen gene. The shuffling was done using *sample* function with *replace = TRUE*. Mean RT of the shuffled genes was calculated for every iteration. For every group we ran 10,000 iterations. The z-score for every group of genes was calculated as (observed RT - mean RT of 10,000 iterations)/standard deviation of RT in 10,000 iterations. The P-values were calculated as a proportion of the iterations with greater or lesser mean RT than the observed RT.

### OLs in BLACK and YELLOW chromatin

Coordinates of BLACK and YELLOW chromatin blocks were obtained from [6]. Gene loci were created from FlyBase genes 5.12 using the leftmost Start and rightmost End positions. Bed tracks were uploaded to Galaxy and coverage for every type of chromatin in every locus was calculated using *Coverage* function in *Operate on Genomic Intervals* section. IGA values for genes [2] were added using *Join two Datasets* function in *Join, Subtract and Group* section. The data were downloaded on a local computer and used to draw the figure in R.

### Shuffling

The statistical significance of overlap was estimated by shuffling using bedtools [28]. UR(B)-regions were randomly shuffled in the *D. melanogaster* genome by shuffleBed (generally 100,000 iterations); the shuffled regions were intersected by intersectBed, and the results were selected using custom awk or bash scripts.

### Localization of OLs to polytene chromosome bands

To map OLs that fail to overlap UR(B)-regions on polytene chromosomes, we used FISH as well as FISH data available from FlyBase [http://flybase.org]. FlyBase data were only used when FISH data for the genes found within several polytene bands around OL were consistent. To assign underreplication status, we used mapping data for weak spots in polytene chromosomes [7], as well as the underreplication mapping data on a physical map of *Drosophila* genome [15], [19].

### FISH mapping of UCR on polytene chromosomes

Flies were raised on standard cornmeal-yeast-agar molasses medium at 22°. Stocks with *SuUR* mutant [17] background where underreplication is suppressed were used, as it allows convenient mapping of FISH signal on polytene chromosomes.

FISH was performed as described in [51]. A set of probes mapping approximately to the middle of every UCR was designed. Genomic DNA was PCR-amplified using the following primers: UCR #98 (5′- AGTGATCGCCGTTGACCGCA-3′ and 5′-ACGATTGCGGGGAGGCCAAA-3′); UCR #598 (5′-AAGTGGCCAATGGGAATACA-3′ and 5′-AGGACCGCAAATAACACAGG-3′); UCR #652 (5′-AGCAGTCGAAATTCGCAAGT-3′ and 5′-TGAATGTCGTTTGGCATTGT-3′);. UCR #808 (5′-CCCTCTTTCCGCGAATCGGC-3′ and 5′-GGCCGCCACTATCTCGTCCA-3′); UCR #872 (5′-TTCAGCCGCTAGGTGTCCCG-3′ and 5′-TGTGCGAGGGTGGCAAGAGA-3′); UCR #1025 (5′-TCGCGTTTCACGCTCGGTTG-3′ and 5′-CCATGTTGCATGGCTCGCTCT-3′); UCR #1354 (5′-GGGGATGAATGGGAAGAGGGGC-3′ and 5′-GCTTTCGGCCCCTTGGGTCA-3′); UCR #1669 (5′-CAATACGTGTGCATCCGTTC-3′ and 5′-GTTCATTAGCCGGTTGCCTA-3′); UCR #1693 (5′-CAATACGTGTGCATCCGTTC-3′ and 5′-GTTCATTAGCCGGTTGCCTA-3′); UCR #1717 (5′-ATCTTCCGATTTCGTCATGC-3′ and 5′-CGTGACGATTGTGGTGAATC-3′); UCR #1744 (5′-AGCTGCATAAAGTCCGGCTA-3′ and 5′-AGAGAGAGAGACCGCACGAC-3′); UCR #1893 (5′-CAGTCGGGATGGCTCTGGCT-3′ and 5′-TGGCGCCCAATGTGAGAGCA-3′); UCR #2533 (5′-ATGCCCCTGTACGCCTGTCC-3′ and 5′-GCCCCTAACGGCTCCCATCT-3′); UCR #2598 (5′-TGCTAGCTCATCGGGAGTTT-3′ and 5′-ATCCTCGGTCTGTGGTTTTG-3′). DNA probes were labeled with biotin-16-dUTP or digoxigenin-11-dUTP (Roche) in random-primed polymerase reaction using Klenow fragment.

Chromosomes were examined using epifluorescence optics (Olympus BX50 microscope) and photographed with CCD Olympus DP50. To localize chromosome regions, we referred to the revised cytological maps of polytene chromosomes of C. B. Bridges (reprinted in [52]).

### Gene density analysis

Gene density within *D. melanogaster* OLs was calculated using UCSC Table Browser. We designed a custom track for OLs in the dm2 genome assembly, where OL borders were defined by the ends of the first and the last gene within a given OL [2]. Altogether, 18 OLs from the following UR(B)-regions were taken into analysis: 7B(OL # 212); 19E (551); 25A1-4(671); 32A1-2(817); 33A1-2(831); 50C1-4(1212); 56AB(1370); 59D1-4(1471); 70A(1839); 70C1-2(1847); 75C1-2(1948); 77E1-4(1995); 84D(2124); 87B(2256); 92D1-4(2424); 92E1-2(2427); 94A1-4(2469); 98C1-2(2615). Gene density within OLs and on OL flanks was calculated as a ratio of the number of genes (multiple isoforms were considered as one gene) to the length of an OL. Sizes of OL flanks were chosen as half the size of the corresponding OL. In *D. melanogaster* and *D. grimshawi*, partial overlap between the flanks was observed, so the total length of flanks was a little lower than that of OLs. This had no effect on the accuracy of gene density analysis, as every gene in the region was counted just once.

To determine gene density in these same OLs and their flanking regions in *D. pseudoobscura*, *D. virilis*, *D. mojavensis* and *D. grimshawi*, we defined OL border coordinates as positions of annotated genes in the beginning and the end of this OL using UCSC Genome Browser. Genome assemblies are available for these species at http://genome.ucsc.edu/, but the coordinates of individual genes (annotated as *D. melanogaster* proteins (BDGP R4/dm2 Proteins) are different from the coordinates used in [2]. So, to make custom tracks of the corresponding OLs and their flanking regions, we determined the coordinates of terminal genes using UCSC Genome Browser. In several cases, OLs fell into groups that belonged to different scaffolds or mapped very far apart (over 1 Mb away from each other). Such OLs were omitted from our analysis for this given species. For these reasons, the analyses performed for *D. mojavensis* and *D. grimshawi* included fewer than 18 OLs. Gene numbers were determined as the number of *D. melanogaster* proteins (multiple isoforms were considered as one gene). Gene density was determined similarly to *D. melanogaster*.

## Supporting Information

Table S1 **Correlation of replication timing (RT) in Kc cells and the IGA score in OLs.**
(XLS)

Table S2 **Correlation of replication timing (RT) in Cl8 cell line and the IGA score in OLs.**
(XLS)

Table S3 **Correlation between IGA score of OLs and chromatin status of genes.**
(XLS)

Table S4 **Ratio of gene densities in OL flanks and in OLs.**
(XLS)

Figure S1 **Replication time in Cl8 cells for genes in OLs with different IGA scores.** Replication score is shown on *x* axes, with +6 corresponding to early replication and −5 denoting late replication. Gene anchor counts are shown on the *y* axes. Replication time for every gene was estimated as an average replication time in Cl8 cells [27] for all probes overlapping the gene. Genes were classified according to the IGA counts in the corresponding OLs. Genes with high IGA counts were combined to get similar-sized groups.
(TIF)

Figure S2 **Numbers of IGAs within OLs – genome-wide and in UR(B)-regions.** *X* axis shows the number of IGAs found in OLs. *Y* axis shows the number of the corresponding OLs in the genome (grey) and within UR(B)-regions (black).
(TIF)

Figure S3 **Different types of overlap between UR(B)-regions and OLs.** Different scales are used for each region. Wide black box denotes an UR(B)-region (name is shown on the left). Colored narrow boxes below correspond to OLs (black: <50 kb; yellow: 50–100 kb; orange: 100–200 kb, red: 200–500 kb, blue: >500 kb). The IGA score is shown under each OL overlapping the UR(B)-region. **A** – pairs of regions, where reciprocal overlap is greater than 80% (the 50C UR(B)-region illustrated here shows 79.8%/93.3% reciprocal overlap with an OL); **B** – reciprocal overlap ranges 65–76%; **C** – UR(B)-region overlaps with a large OL, but the extent of overlap is below 65%; **D** – UR(B)-region overlaps with several smaller OLs, neither of which appears to be dominant length-wise. Pairs of neighboring UR(B)-regions that are covered by a single common OL are displayed as a single UR(B)-region (further details in the main text). Pairs of UR(B)-regions are denoted as follows: (19E1-2+19E3-4) = 19E; (56A1-2+56B1-2) = 56AB; (64C1-2+64C3-4) = 64C; (70A1-2+70A4-5) = 70A; (84D3-4+84D9-10) = 84D; (87B1-2+87B4-5) = 87B; (89A1-2+89A8-9) = 89A.
(TIF)

Figure S4 **Mapping of UCRs in** ***Drosophila*** **salivary gland polytene chromosomes.** UCR ID number is shown on top of each image. Cytology position of FISH signal is indicated in parentheses.
(TIF)

Text S1 **Data obtained under OLC and GOO synteny definition** [**2**] **.**
(PDF)
