# Supplementary figures and images for: Late Replication Domains Are Evolutionary Conserved in the Drosophila Genome
Source: PLoS One. 2013 Dec 31;8(12):e83319. doi: 10.1371/journal.pone.0083319 (PMC3877026; doi:10.1371/journal.pone.0083319)

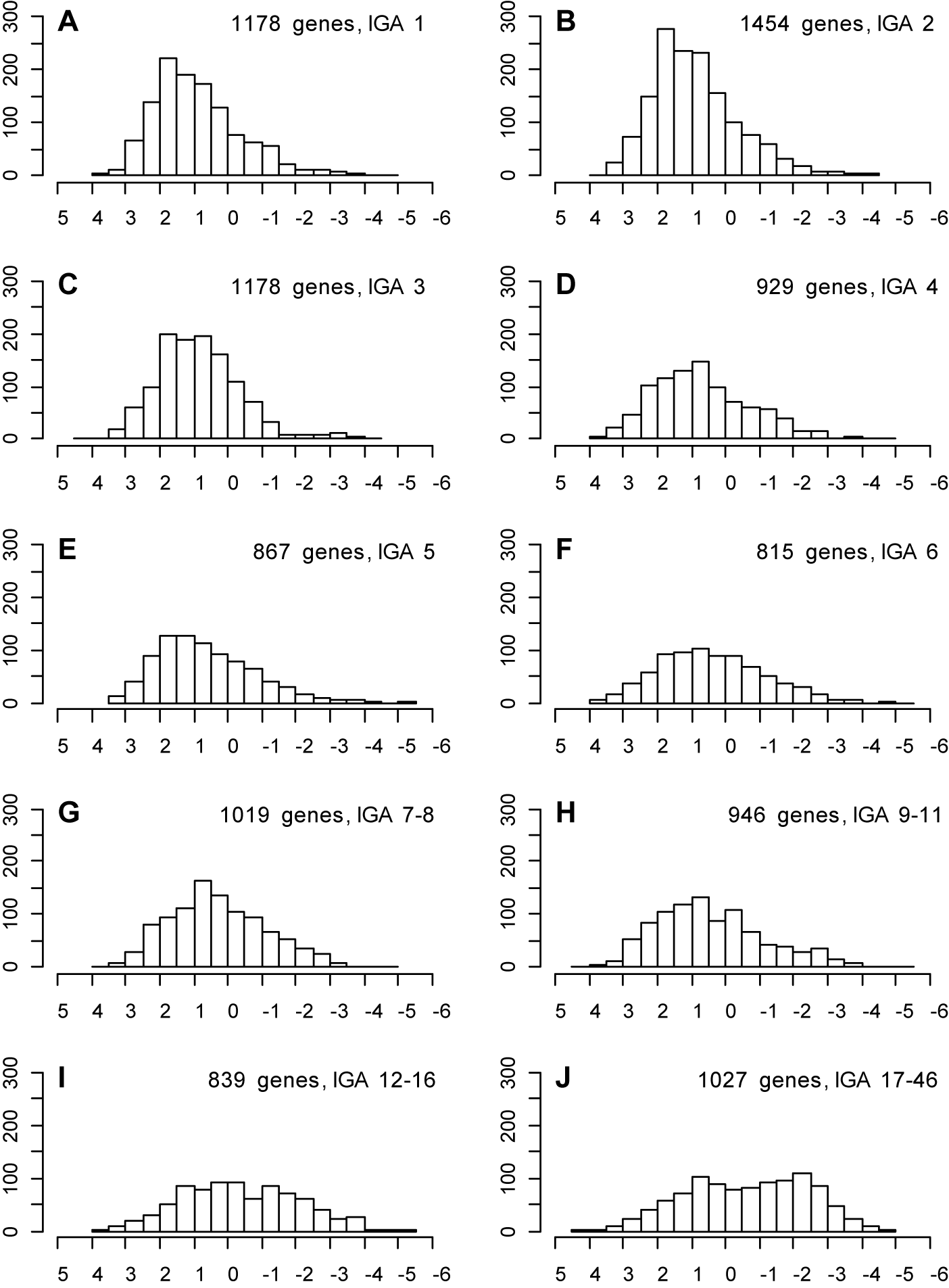

Supplement: Figure S1 — Replication time in Cl8 cells for genes in OLs with different IGA scores. Replication score is shown on x axes, with +6 corresponding to early replication and −5 denoting late replication. Gene anchor counts are shown on the y axes. Replication time for every gene was estimated as an average replication time in Cl8 cells [27] for all probes overlapping the gene. Genes were classified according to the IGA counts in the corresponding OLs. Genes with high IGA counts were combined to get similar-sized groups. (TIF) [file pone.0083319.s005.tif]

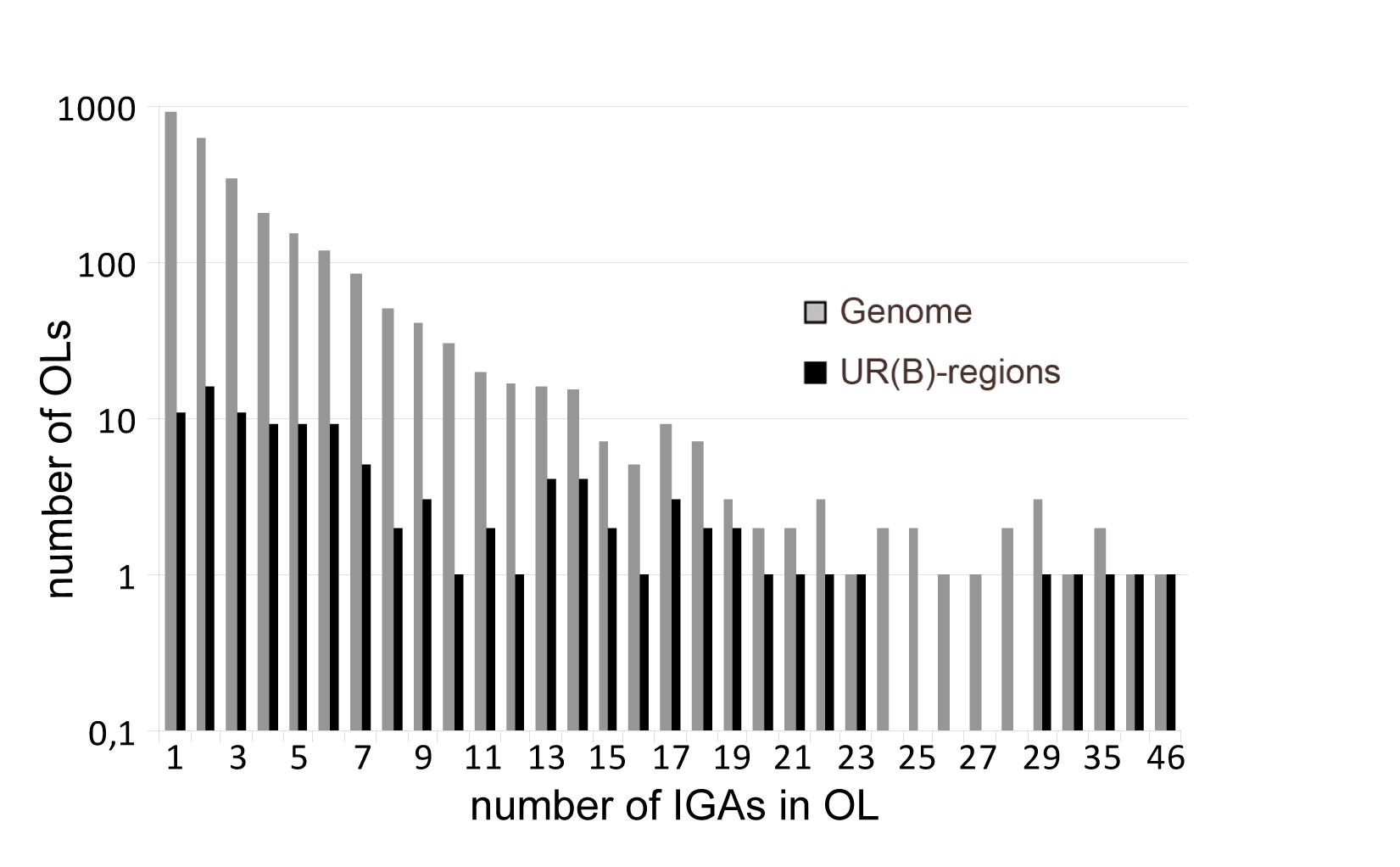

Supplement: Figure S2 — Numbers of IGAs within OLs – genome-wide and in UR(B)-regions. X axis shows the number of IGAs found in OLs. Y axis shows the number of the corresponding OLs in the genome (grey) and within UR(B)-regions (black). (TIF) [file pone.0083319.s006.tif]

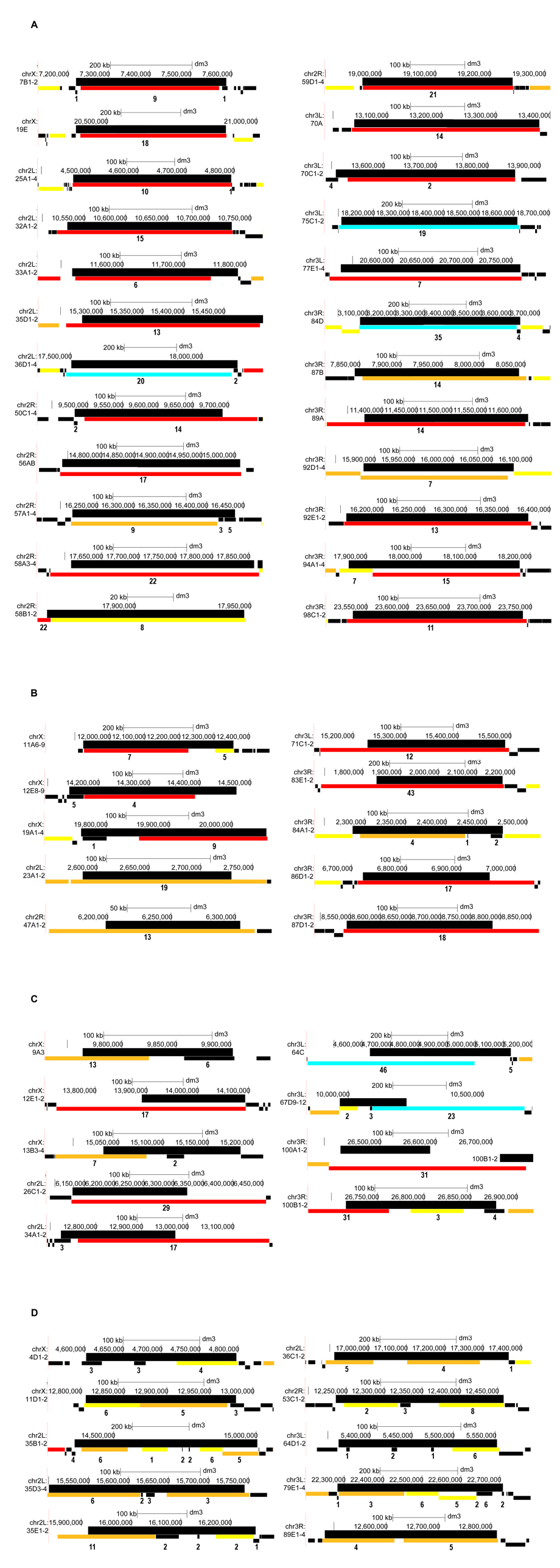

Supplement: Figure S3 — Different types of overlap between UR(B)-regions and OLs. Different scales are used for each region. Wide black box denotes an UR(B)-region (name is shown on the left). Colored narrow boxes below correspond to OLs (black: <50 kb; yellow: 50–100 kb; orange: 100–200 kb, red: 200–500 kb, blue: >500 kb). The IGA score is shown under each OL overlapping the UR(B)-region. A – pairs of regions, where reciprocal overlap is greater than 80% (the 50C UR(B)-region illustrated here shows 79.8%/93.3% reciprocal overlap with an OL); B – reciprocal overlap ranges 65–76%; C – UR(B)-region overlaps with a large OL, but the extent of overlap is below 65%; D – UR(B)-region overlaps with several smaller OLs, neither of which appears to be dominant length-wise. Pairs of neighboring UR(B)-regions that are covered by a single common OL are displayed as a single UR(B)-region (further details in the main text). Pairs of UR(B)-regions are denoted as follows: (19E1-2+19E3-4) = 19E; (56A1-2+56B1-2) = 56AB; (64C1-2+64C3-4) = 64C; (70A1-2+70A4-5) = 70A; (84D3-4+84D9-10) = 84D; (87B1-2+87B4-5) = 87B; (89A1-2+89A8-9) = 89A. (TIF) [file pone.0083319.s007.tif]

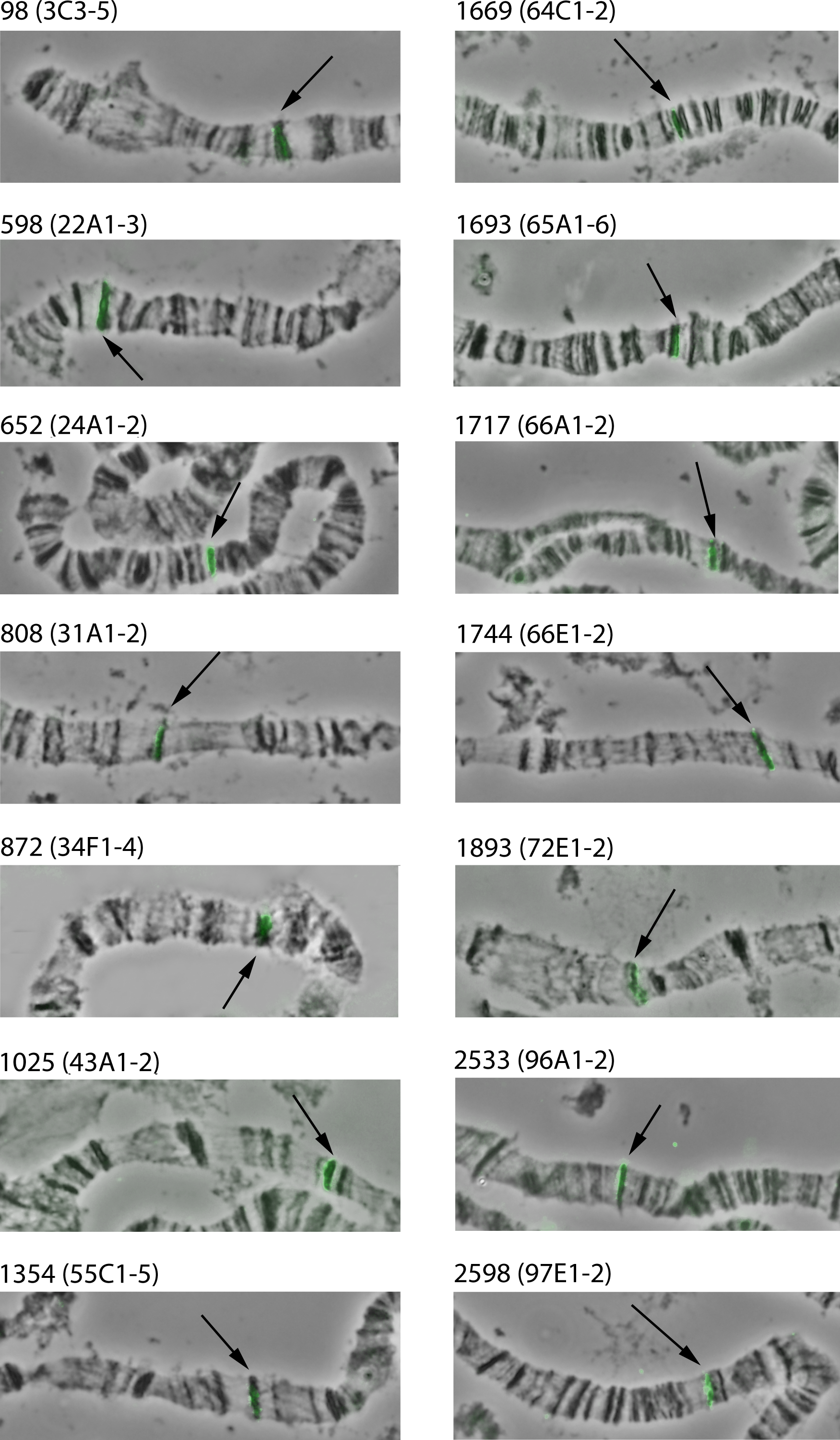

Supplement: Figure S4 — Mapping of UCRs in Drosophila salivary gland polytene chromosomes. UCR ID number is shown on top of each image. Cytology position of FISH signal is indicated in parentheses. (TIF) [file pone.0083319.s008.tif]
