# Supplementary material for: Late Replication Domains Are Evolutionary Conserved in the Drosophila Genome
Source: PLoS One. 2013 Dec 31;8(12):e83319. doi: 10.1371/journal.pone.0083319 (PMC3877026; doi:10.1371/journal.pone.0083319)
Supplement: Text S1 — Data obtained under OLC and GOO synteny definition [2] . (PDF) [file pone.0083319.s009.pdf]

Supplementary text

All the data presented in this paper were obtained under GO synteny definition (conservation of gene order regardless of gene orientation). Yet, we demonstrate that our conclusions regarding the matching positions of OLs and UR(B)-regions also hold true when two more synteny definitions proposed by von Grotthuss et al. [2] are used, namely GOO (conservation of gene order and orientation) and OLC (overall local contiguity) where gene shuffling within an OL is allowed. Below we present the numbers obtained under GOO and OLC definitions.

1. UR(B)-regions overlap with 188 GOO OLs, 2.1-fold less than the average observed in the shuffling simulation (400; 100,000 iterations; P-value < 1E-5). Just 86 OLC OLs overlap with the UR(B)-regions, 3.9-fold less than the shuffled control average 339 (100,000 iterations; P-value < 1E-5). Thus, similarly to GO-, both GOO- and OLC-defined OLs display pronounced decrease in synteny disruption within UR(B)-regions.

2. Under OLC and GOO definitions, reciprocal overlap of greater than 80% between UR(B)-region and a single OL is observed in 21 and 9 out of 60 regions, respectively. This is 9.5- and 5.3-fold more than expected (10,000 shuffling iterations, P-value < 1E-4). Therefore, alternative definitions of synteny similarly produce significant overlap between UR(B)-regions and OLs.

3. Under both OLC and GOO definitions, OLs are observed to be generally biased towards larger sizes. Given that GOO definition is strict and takes into account gene orientation, UR(B)-regions now include more of the short OLs (<10 kb), yet the percentage of long OLs (>100 kb) is well above the expected value. The bar plot shows the percentage of OLs (%) of a given size observed in UR(B)-regions (blue bars) and the shuffled value simulated from a randomized set of UR(B)-regions (red bars) – as obtained for OLC (Figure A) and GOO (Figure B) definitions of synteny. P-values are given on top of the bars where expected and observed values are statistically different.

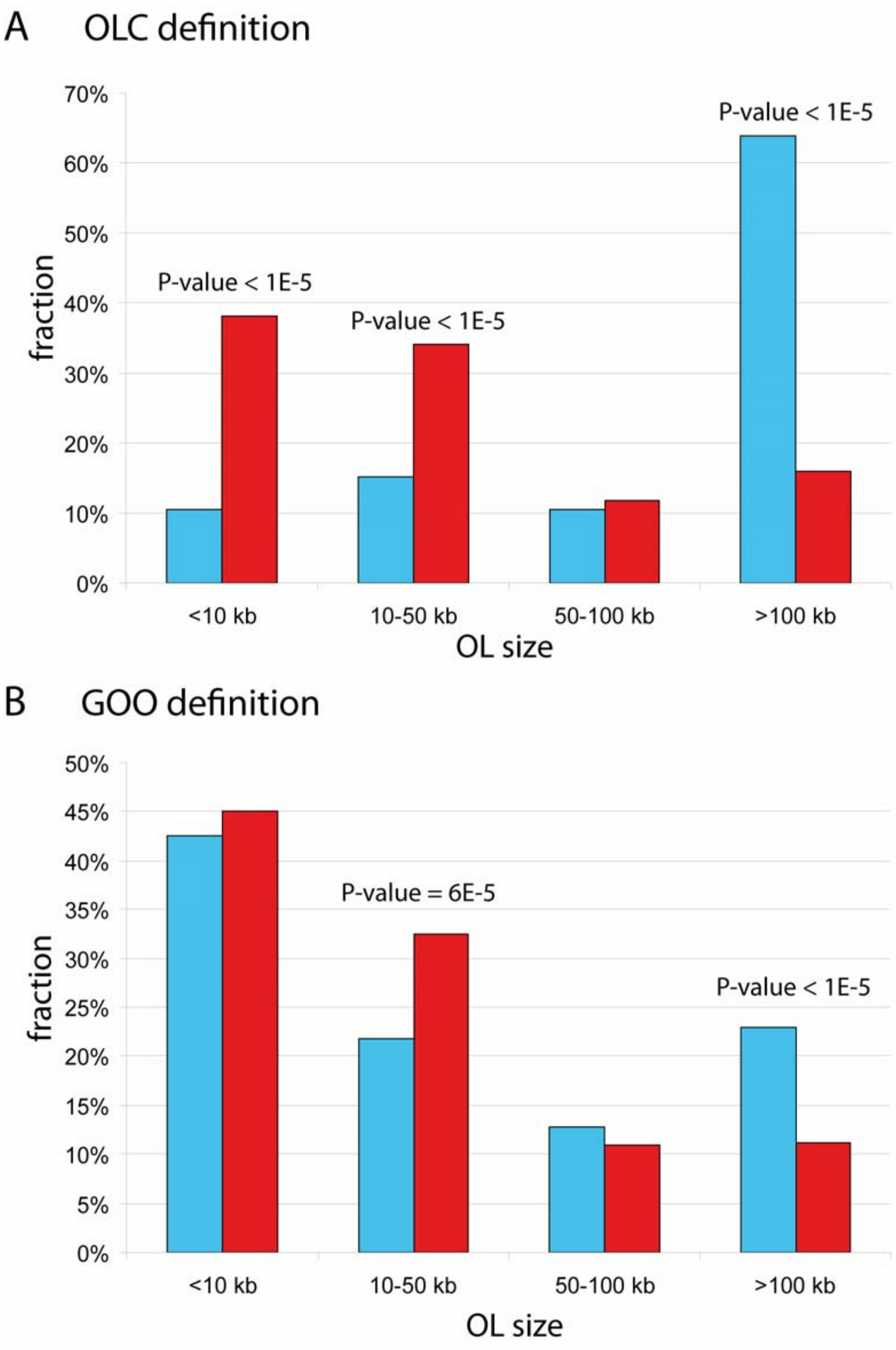
A
OLC definition
70%
60%
50%
40%
30%
20%
10%
0%
fraction
P-value < 1E-5
P-value < 1E-5
P-value < 1E-5
<10 kb
10-50 kb
50-100 kb
>100 kb
OL size
B
GOO definition
50%
45%
40%
35%
30%
25%
20%
15%
10%
5%
0%
fraction
P-value = 6E-5
P-value < 1E-5
<10 kb
10-50 kb
50-100 kb
>100 kb
OL size
